# Combined islet and kidney xenotransplantation for diabetic nephropathy: an update in ongoing research for a clinically relevant application of porcine islet transplantation

**DOI:** 10.3389/fimmu.2024.1351717

**Published:** 2024-02-27

**Authors:** Daniel L. Eisenson, Hayato Iwase, Weili Chen, Yu Hisadome, Wanxing Cui, Michelle R. Santillan, Alexander C. Schulick, Du Gu, Amanda Maxwell, Kristy Koenig, Zhaoli Sun, Daniel Warren, Kazuhiko Yamada

**Affiliations:** ^1^ Department of Surgery, The Johns Hopkins University School of Medicine, Baltimore, MD, United States; ^2^ Cell Therapy and Manufacturing, Medstar Georgetown University Hospital, Washington DC, United States; ^3^ Research Animal Resources, The Johns Hopkins University School of Medicine, Baltimore, MD, United States

**Keywords:** islet xenotransplantation, islet-kidney, xenogeneic immune response, tolerance, xenotransplantation

## Abstract

Combined islet and kidney xenotransplantation for the treatment of diabetic nephropathy represents a compelling and increasingly relevant therapeutic possibility for an ever-growing number of patients who would benefit from both durable renal replacement and cure of the underlying cause of their renal insufficiency: diabetes. Here we briefly review immune barriers to islet transplantation, highlight preclinical progress in the field, and summarize our experience with combined islet and kidney xenotransplantation, including both challenges with islet-kidney composite grafts as well as our recent success with sequential kidney followed by islet xenotransplantation in a pig-to-baboon model.

## Introduction

Diabetes is a leading cause of both cardiovascular disease and end stage renal disease (ESRD), and incidence is increasing across the country and across the globe (1). Human islet transplantation is an effective treatment for diabetic patients but requires lifelong immunosuppression: prospective islet transplant recipients must weigh the risks of immunosuppression against the short- and long-term complications of diabetes. Patients with diabetic nephropathy represent a unique – and growing – population that would benefit from both islet and kidney transplantation. Indeed, the favorable risk-to-benefit considerations of combined islet and kidney transplantation in this population inspired recent promising clinical studies in islet after kidney transplantation led by the Clinical Islet Transplantation (CIT) Consortium (2). However, at present these procedures are rare, due, in part, to a shortage of deceased donor organs (3). Xenotransplantation using organs derived from pigs may overcome this organ shortage and allow for broader application of combined islet and kidney transplantation.

The past several years have seen enormous progress in the field of xenotransplantation, with advances in gene-editing and immunosuppression leading to long-term survivals of both kidney and heart xenografts in pig-to-nonhuman primate (NHP) studies (4–7), as well as early studies (preclinical and clinical) in humans (8–10). Clinical translation of porcine islet transplantation predated these recent successes in solid organ xenotransplantation, with encouraging pig-to-NHP studies leading to several small clinical studies using porcine islets in humans (11–21). However, results of these early studies in clinical islet xenotransplantation have been mixed. While these differences between outcomes of preclinical and clinical xenogeneic islet transplantation may be partly explained by differences in the immunosuppression regimens used in the clinical trials – notably, CD40/CD40L costimulatory blockade, which has been critical to success in most preclinical studies, was not utilized – further trials have been limited by more recent consensus guidelines outlining an international framework to promote standardized clinical translation of pig-to-human islet transplantation from source pig development and manufacturing to patient monitoring (22).

Moreover, in the many years since these clinical studies in islet xenotransplantation were conducted, the landscape of diabetes management has changed. Patients with diabetes have other options for durable disease management. Innovations in glucose monitoring and the rapid development of hybrid closed-loop insulin delivery systems have improved quality of life for patients living with diabetes (23), and ongoing clinical trials of novel stem-cell derived islet cell therapy have published early and highly promising results (24). However, porcine islet xenotransplantation remains a compelling therapeutic possibility for patients with diabetic nephropathy who need both kidney and islet replacement. In these patients, there are minimal added risks associated with islet transplantation, as these patients are already on immunosuppression for their kidney grafts; in fact, islets may help protect against premature kidney graft loss associated with diabetes (25) as well as improve long-term vascular diabetic outcomes (26). Here, we will briefly highlight immunologic barriers in porcine islet transplantation, chronicle preclinical progress in the field, and summarize our own experience in combined islet and kidney transplantation, using both 1) vascularized islets in an islet-kidney composite graft, and 2) our more recent strategy of sequential kidney followed by islet xenotransplantation.

## Immunologic barriers in pig-to-primate islet xenotransplantation

Porcine xenografts, including pig islets, elicit robust immune responses in humans. These responses involve both innate barriers (27) – including preformed natural antibodies (Nabs) and species incompatibilities in complement and coagulation systems leading to dysregulation – and adaptive immune components (reviewed in (28)). As with transplantation of solid organs, humoral immunity remains a key obstacle to long-term xenograft survival, and T cell-targeted immunosuppression strategies have been critical for prolonging islet survival (20, 29),. Unlike transplantation of other organs, however, transplanted islets also trigger an immediate inflammatory response, known as instant blood-mediated inflammatory reaction (IBMIR) (30, 31), related to expression of tissue factor on islets and leading to activation of innate responses that subsequently consume islets (32, 33). Although IBMIR is seen in auto- and allogeneic islet transplantation, greater immune barriers in xenotransplantation may lead to more pronounced islet losses (34, 35) as high as 70% in some studies (36).

## Preclinical progress in porcine islet xenotransplantation: encapsulation and source pig genetic modifications

Various strategies have been developed to overcome these short- and long-term immunologic hurdles, including islet encapsulation and source pig genetic modifications – both of which are intended to reduce the immunogenicity of the porcine islets.

Broadly, islet encapsulation technologies include microencapsulation of islets in alginate matrix, and macro-encapsulation of immobilized islets in bi-layered PTFE with a common oxygenation chamber (37). This microencapsulation technique successfully reversed diabetes for up to six months in preclinical studies of rhesus macaques (38), and was subsequently used in two nationally regulated clinical studies of porcine islet xenotransplantation in New Zealand and Argentina. Follow-up studies confirmed modest clinical benefit including reduction in HbA1c, hospitalization, and severe hypoglycemic and/or hyperglycemic events (21, 39 *)* The key advantage of these technologies is that encapsulation may protect islets from the recipient immune system and obviate the need for immunosuppression; whereas islet transplantation alone is currently reserved for patients with hypoglycemic unawareness due to the morbidity of immunosuppression, transplantation of encapsulated islets without immunosuppression may tilt the risk-benefit ratio in favor of islet transplantation for cure of diabetes. Still, important hurdles remain in broader clinical application of this technology including variable recipient immune responses to the encapsulation material, which may lead to fibrosis of encapsulated grafts.

Source pig genetic modification is another strategy to overcome innate immune barriers and can be divided into two major categories: elimination of carbohydrate antigens that are targets of preformed antibodies, and correction of species incompatibilities. In solid organ xenotransplantation, preformed antibody binding leads to hyperacute rejection of graft; in free islet xenotransplantation, preformed antibody binding leads to an amplified IBMIR with islet loss (40). While elimination of targets of preformed Nabs (particularly elimination of *α-gal* with creation of α-1,3 Galactosyl transferase gene knockout or GalTKO source pigs) has been essential for successful pig-to-NHP heart and kidney xenotransplantation (4, 41, 42), the impact of using GalTKO source pigs on xenograft survival in islet transplantation is less conclusive, which may be a function of changes in α-gal expression with islet maturation (43–45). Similarly, correcting for species incompatibilities between porcine and primate complement regulatory systems through individual insertion of human complement regulatory proteins may not significantly reduce the incidence of IBMIR (44). However, combining carbohydrate antigen gene knockouts with complement regulatory transgenes proves additive: xenogeneic islets from GalTKO.hCD55.hCD59 and GalTKO.hCD39.hCD46 source pigs demonstrated reduced islet loss and attenuated IBMIR (15, 16). More recently, islets derived from neonatal GalTKO.hCD55.hCD59 source pigs demonstrated cure of diabetes with >1 year of insulin independence in the stringent pig-to-baboon model (46).

## Combined kidney and islet xenotransplantation to broaden clinical applicability of porcine islet xenotransplantation

Marked improvements in diabetes management and emerging therapies have changed the risk-benefit calculus associated with islet transplantation more broadly, and porcine islet xenotransplantation in particular. As described in the preceding section, encapsulation technologies – which may allow for durable glucose control without immunosuppression – remain one relevant application for porcine islet xenotransplantation. Another relevant strategy is combining porcine islet xenotransplantation with solid organ xenotransplantation. This strategy has already been employed with success by the CIT consortium treating diabetic nephropathy with islet transplantation after kidney transplantation, but broader application is limited by the shortage of deceased donor organs. The following sections detail our preclinical experience with combined islet and kidney transplantation, including both composite islet-kidney transplantation as well as kidney-first sequential islet and kidney xenotransplantation.

## Combined islet and kidney xenotransplantation: our experience with composite islet-kidney xenotransplantation

### Composite islet-kidney transplantation for cure of diabetic nephropathy: concept and supporting allogeneic data

**Figure 1:**
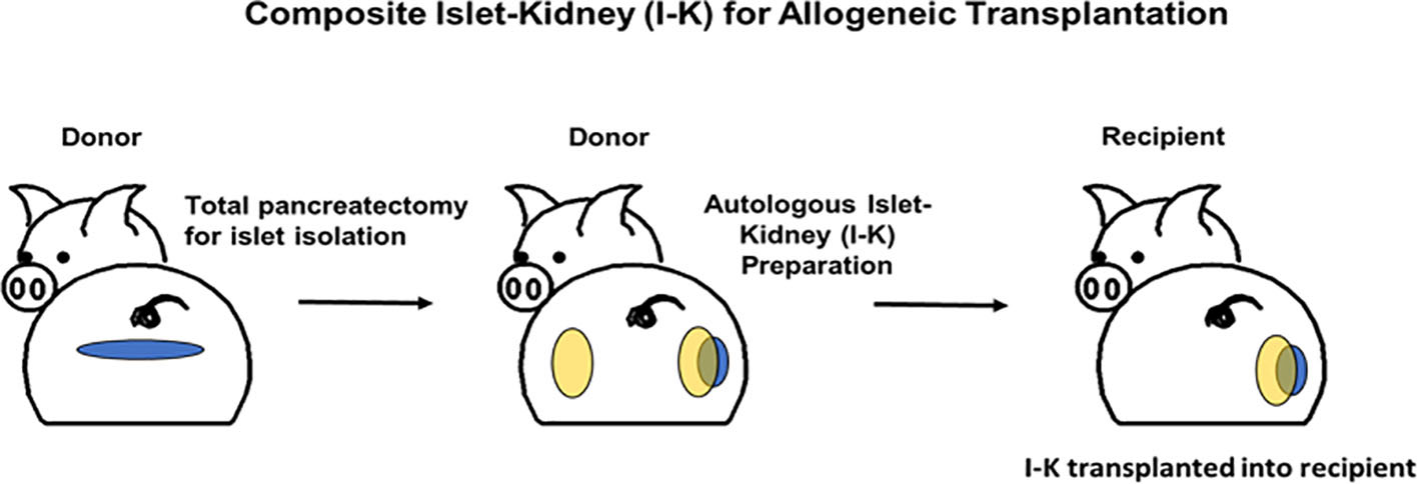
Schematic diagrams of preparation of a composite islet-kidney graft in donor and allogeneic composite islet-kidney transplantation in a recipient.

As detailed above, xenogeneic islets are susceptible to destruction by both innate and adaptive mechanisms. The senior author of this review has demonstrated that transplanting pre-vascularized islets as part of a composite organ protects islets from innate immune destruction by circumventing the typical pathway that triggers IBMIR (47–49). We have previously reported successful preparation of composite islet-kidney (I-K) grafts which maintained normoglycemia and normal renal function after transplantation in pig-to-pig and nonhuman primate allogeneic transplantation models (**Figure 1**). Islets are isolated and pre-vascularized under autologous renal capsule, with subsequent transplantation of composite I-K graft (50). Preclinical allotransplantation studies in both pigs and in NHPs have demonstrated that this procedure preserves islets, likely by limiting innate immune destruction: diabetes is cured in animals who undergo composite I-K transplantation, while animals who undergo conventional free islet injection with the same islet equivalents (IEQs) remain insulin dependent (51, 52). Additional preclinical studies have demonstrated further improvements in islet yield and function with islet protective strategies including siRNA silencing of apoptotic genes (53). However, practical challenges have limited successful translation of this composite organ strategy for cure of diabetes and kidney failure in pig-to-NHP transplantation.

### Challenges with translation of allogeneic results to xenogeneic pig-to-non-human primate model

While I-K composite organs are ideally created in the same animal with autologous pig islets transplanted under autologous renal capsule, size constraints in our NHP recipient prevent successful I-K transplantation using a single source pig: a small (<30kg) source pig is needed for successful pig-to-NHP kidney transplantation, while a large (>60kg) source pig is needed in order to obtain sufficient islets for reversal of diabetes. This is a limitation primarily in our preclinical pig-to-NHP model, as larger kidneys from size matched >60kg pigs will likely be appropriate for human adult recipients. Nevertheless, overcoming this experimental constraint is critical to demonstration of composite I-K success. Our own attempts to isolate islets from juvenile source pig pancreases recapitulated the work of other investigators, confirming low islet-equivalent yield from juvenile pigs (54, 55). Accordingly, we elected to use two different source pigs for composite organ creation: large pigs would be used for islets, pre-vascularized prior to transplantation under the renal capsule of a smaller pig.

Using different source pigs for I-K composite organ creation introduced other challenges. Allogeneic islets from one source pig transplanted under the renal capsule of another source pig are vulnerable to recipient source pig immune responses, as with any allogeneic transplant. Strategies to mitigate these responses include 1) use of related pairs (cloned, inbred, or MHC-matched), and 2) minimization of the islet pre-vascularization period under allogeneic renal capsule.

### Using related pairs for composite islet-kidney creation

Although the use of genetically identical (cloned) pairs for islet-kidney creation would be ideal, previous experiments have demonstrated long-term survival of skin and heart grafts without immunosuppression in highly inbred swine (56). Indeed, inbred animals, defined by co-ancestry >0.9, accepted allogeneic skin grafts for >340 days and accepted allogeneic heart grafts for >265 days without immunosuppression. MHC-matched pairs (co-ancestry >0.75) also allowed for acceptance of kidney grafts without immunosuppression, although hearts and islets were not accepted (50, 56, 57). Over the last three years, we have optimized composite I-K preparation using MHC-matched source pigs. As opposed to autologous I-K preparation, successful allogeneic preparation requires immunosuppression with both high dose tacrolimus and MMF. I-K preparation may be further optimized with reduction in pre-vascularization period from 6 weeks to 2 weeks. Still, it remains unclear whether I-K preparation in MHC-matched pairs preserves sufficient islets for reversal of diabetes in a xenogeneic recipient. We plan to revisit the composite I-K strategy in xenotransplantation when cloned pigs or highly inbred GalTKO pigs are available for these experiments.

## Combined islet and kidney xenotransplantation: recent success with sequential kidney followed by islet xenotransplantation

Definitive evaluation of composite I-K transplantation in a pig-to-NHP model also requires a control: independent kidney and free islet transplantation. Negative controls were present in previous studies of composite IK technologies – in pig-to-pig, baboon-to-baboon, and macaque-to-macaque models – and demonstrated preservation of islets with composite I-K transplantation across allogeneic barriers. In the past year, due to lack of inbred or cloned source pigs, we tested an alternative strategy for combined islet and kidney transplantation across a xenogeneic barrier that would also serve as a control of the composite I-K strategy. This alternative approach involves delayed islet transplantation after kidney and vascularized thymus transplantation (role of vascularized thymus transplantation in the induction of tolerance across xenogeneic barriers reviewed in (28)), using a recipient size-matched kidney and thymus source pig, as well as a large source pig for islets. Notably this approach (without thymus co-transplantation) is also similar to recent work in human islet-after-kidney transplantation conducted by the CIT consortium. Although additional cases are required, we have achieved reversal of diabetes and life-supporting renal function for 180 days with this kidney-first sequential islet and kidney xenotransplantation (52). To our knowledge, this is the first demonstration of maintenance of durable normoglycemia and stable creatinine with porcine kidney and islets in a diabetic and life-supporting pig-to-baboon combined kidney, vascularized thymus and islet xenotransplantation model. These preliminary results were recently presented at the International Xenotransplantation Association Congress (San Diego, 2023), and are described in detail in the following sections:

### Methods: source pigs, recipient baboons, immunosuppression regimen, and transplantation procedures

In this experiment, we used two GalTKO.hCD55 source pigs from the National Swine Resource and Research Center (*Sus scrofa domesticus*, source: University of Missouri-Columbia, Columbia, MO) and one baboon recipient from the National Research and Resources Program (MD Anderson, Houston TX). Baboon recipient underwent B and T cell depletion with rituximab and rabbit anti-thymocyte globulin, followed by maintenance immunosuppression with anti-CD40 mAb (Nonhuman Primate Reagent Resource, University of Massachusetts Medical School, Worcester, MA). The baboon received kidney and vascularized thymic lobe grafts from a GalTKO.hCD55 (11.7kg) source pig on POD 0 with bilateral native nephrectomy. Diabetes was subsequently induced with streptozosin (STZ. 100mg/kg on POD 5, 50mg/kg on POD 9). After confirmation of diabetes, baboon underwent free islet transplantation into the portal vein, with islets isolated from unrelated GalTKO.hCD55 (95kg) source pig. Islet isolation was performed as previously described (58) and yielded 101K islet equivalents (IEQs) and 194K islet particle number (IPN). All animals were used in compliance with guidelines provided by the Animal Care and Use Committee at The Johns Hopkins University School of Medicine.

### Results: islet-after-kidney pig-to-baboon xenotransplantation cures diabetes and renal insufficiency

Both renal insufficiency and diabetes were cured with kidney-first sequential islet and kidney xenotransplantation. The baboon recipient maintained normal serum creatinine with no evidence of rejection for six months following kidney and islet transplant but was euthanized due to sepsis related to pyelonephritis in setting of stent occlusion on POD180. Immediately after islet transplantation, hyperglycemia was reversed with normalization of blood sugars from >250mg/dL (pre-transplant) to 80-110 mg/dL. Porcine islets functioned and maintained normal BG levels without any exogenous insulin treatment throughout the recipient’s postoperative course. Post-mortem evaluation of liver confirmed presence of insulin-staining islets.

### Discussion: timing of sequential transplants and immunomodulatory strategies may be important for success of islet-after-kidney xenotransplantation

As referenced above (see *Preclinical progress in porcine islet xenotransplantation*), investigators have recently achieved cure of diabetes in baboons using pooled islets from neonatal genetically modified pig donors (46). However, this required an average of 14 neonatal pancreases (70 piglets for 5 baboon recipients). In our model, we have achieved normoglycemia using islets derived from a single source adult pig with an administered islet mass of 12,500 IEQ/kg. Of note, this is within range though slightly less than was required in the recent clinical islet-after-kidney transplantation studies where successful islet transplants averaged >16,000 IEQ/kg (2).

One reason for the success of free islet transplantation in this model may be timing of sequential transplants: kidney-first transplantation promotes absorption of anti-pig antibodies, likely reducing IBMIR following islet transplantation, corresponding to reduced loss of islets. This may have enabled durable reversal of diabetes with fewer islet equivalents as compared with clinical islet-after-kidney transplantation. Indeed, the possible antibody absorption benefits of sequential transplant timing is less clear in the clinical islet-after-kidney studies, where islet transplantation occurred well after index kidney transplantation.

Lastly, adjunctive immunomodulatory strategies may also have played a role in the durable xenograft survival in this case. This animal received vascularized thymic lobe (VTL) graft co-transplantation from the kidney source pig, which has been shown to prolong xenograft survival in pig-to-baboon renal xenotransplantation (reviewed in (28)). Interferon gamma (IFN-γ) enzyme-linked immunosorbent spot (ELISpot) assay was performed to assess the potential immunomodulatory effect of VTL co-transplantation in this case. ELISpot assay at POD 180 demonstrated pig-specific unresponsiveness, suggesting that co-transplanted VTL graft may promote immunomodulatory effects. Further studies will clarify the mechanisms of *in vitro* unresponsiveness (59).

Additional cases are needed to replicate this work, but these encouraging results indicate that our negative control strategy, sequential kidney followed by islet xenotransplantation may reverse diabetes and renal insufficiency.

## Porcine islet xenotransplantation: the best path forward may be dual indication transplantation

Porcine islet xenotransplantation is one promising strategy for cure of diabetes among an evolving landscape of emerging therapies in diabetes management. While islet-alone xenotransplantation strategies continue to show improvement with source pig genetic modifications and refinements to immunosuppression regimens, approaches like encapsulation which allow for reversal of diabetes without immunosuppression may be more clinically relevant. Porcine islet xenotransplantation, in conjunction with kidney xenotransplantation, remains a particularly compelling therapeutic possibility for patients with diabetic nephropathy who require both kidney and islet replacement, and who have already committed to immunosuppression for their kidney grafts. Composite islet-kidney transplantation has proven challenging in xenogeneic preclinical models; however, preliminary studies in islet-after-kidney xenotransplantation are promising and may point to a path forward with combined islet and kidney transplantation for diabetic nephropathy.

## Author contributions

DE: Conceptualization, Formal Analysis, Investigation, Methodology, Writing – original draft. HI: Data curation, Formal Analysis, Investigation, Writing – review & editing. WLC: Formal Analysis, Methodology, Writing – review & editing. YH: Formal Analysis, Investigation, Writing – review & editing. WXC: Methodology, Writing – review & editing. MS: Formal Analysis, Investigation, Writing – review & editing. AS: Investigation, Writing – review & editing. DG: Methodology, Writing – review & editing. AM: Investigation, Writing – review & editing. KK: Methodology, Project administration, Writing – review & editing. ZS: Methodology, Supervision, Writing – review & editing. DW: Investigation, Methodology, Project administration, Writing – review & editing. KY: Conceptualization, Investigation, Methodology, Project administration, Supervision, Writing – review & editing.
